# Prognostic and clinical significance of long non-coding RNA SNHG12 expression in various cancers

**DOI:** 10.1080/21655979.2020.1831361

**Published:** 2020-10-30

**Authors:** Chenghao Zhang, Xiaolei Ren, Wenchao Zhang, Lile He, Lin Qi, Ruiqi Chen, Chao Tu, Zhihong Li

**Affiliations:** a Department of Orthopedics, the Second Xiangya Hospital, Central South University, Changsha, China; b Hunan Key Laboratory of Tumor Models and Individualized Medicine, the Second Xiangya Hospital, Central South University, Changsha, China

**Keywords:** LncRNA, cancer, sarcoma, SNHG12, prognosis

## Abstract

Recently, increasing studies suggested that lncRNA SNHG12 was aberrantly expressed in kinds of cancers. However, definite prognostic value of SNHG12 remains unclear. We conducted this meta-analysis to evaluate the association between SNHG12 expression level and cancer prognosis. A literature retrieval was conducted by searching kinds of databases. The meta-analysis was performed by using Revman 5.2 and Stata 12.0 software. Besides, The Cancer Genome Atlas dataset was analyzed to validate the results in our meta-analysis via using Gene Expression Profiling Interactive Analysis. The pooled results showed that high SNHG12 expression significantly indicated worse overall survival and recurrence-free survival. Tumor type, sample size, survival analysis method, and cutoff value did not alter SNHG12 prognosis value according to stratified analysis results. Additionally, higher expression of SNHG12 suggested unfavorable clinicopathological outcomes including larger tumor size, lymph node metastasis, distant metastasis, and advanced clinical stage. Online cross-validation in TCGA dataset further indicated that cancer patients with upregulated SNHG12 expression had worse overall survival and disease-free survival. Therefore, elevated SNHG12 expression was associated with poor survival and unfavorable clinical outcomes in various cancers, and therefore might be a potential prognostic biomarker in human cancers.

**Abbreviations** Akt: protein kinase B; CESC: cervical squamous cell carcinoma and endocervical adenocarcinoma; ceRNA: competitive endogenous RNA; CNKI: China National Knowledge Infrastructure; CI: confidence interval; CCNE1: cyclin E1; COAD: colon adenocarcinoma; DM: distant metastasis; DFS: disease-free survival; EMT: epithelial–mesenchymal transition; FISH: fluorescence in situ hybridization; FIGO: the International Federation of Gynecology and Obstetrics; GEPIA: Gene Expression Profiling Interactive Analysis; HR: hazard ratio; HIFα: hypoxia-inducible factor 1 α; KIRC: kidney renal clear cell carcinoma; KIRP: kidney renal papillary cell carcinoma; LIHC: hepatocellular carcinoma; LNM: lymph node metastasis; mTOR: mechanistic target of rapamycin kinase; MMP-9: matrix metalloproteinase 9; MCL1: myeloid cell leukemia 1; MLK3: mixed-lineage protein kinase 3; N/A: not available; NOS: Newcastle-Ottawa Scale; OR: odd ratio; OS: overall survival; PSA: prostate-specific antigen; PI3K: phosphoinositide 3-kinase; qRT-PCR: quantitative real-time polymerase chain reaction; READ: rectum adenocarcinoma; RFS: recurrence-free survival; SARC: sarcoma; SNHG12: small nucleolar RNA host gene 12; STAT3: signal transducer and activator of transcription 3; SOX4: SRY-box transcription factor 4; SOX5: SRY-box transcription factor 5; STAD: stomach adenocarcinoma; TCGA: The Cancer Genome Atlas; TNM: tumor node metastasis; WWP1: WW domain-containing E3 ubiquitin protein ligase 1; WHO grade: World Health Organization grade; ZEB2: zinc finger E-box-binding homeobox 2

## Introduction

Nowadays, cancer has been a leading cause of mortality worldwide and has brought huge burdens to patients, families and society [1]. Despite numerous achievements in surgical resection, radiotherapy, chemotherapy, and immunotherapy [2–4], cancer patients suffer from disappointing survival outcomes and life quality, especially for patients with advanced clinical stage or metastatic cancer [5]. The insufficiency of effective prognosis biomarkers is supposed to be a crucial reason for this. Consequently, there remains a need to identify novel prognosis biomarkers for predicting cancer prognosis and therapeutic efficacy [6].

**Figure:**
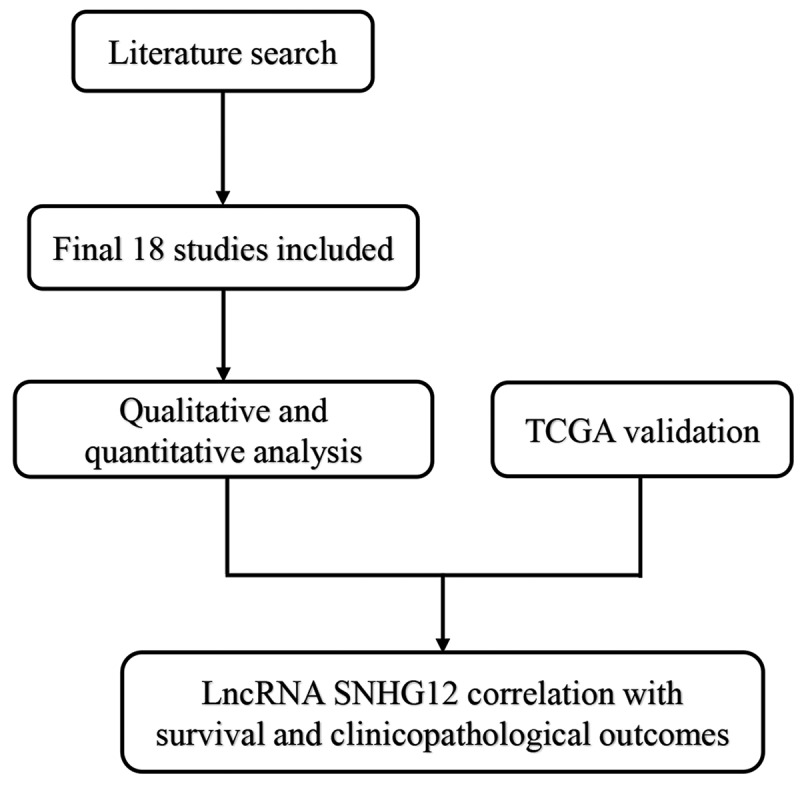

Long noncoding RNAs (lncRNAs) is a class of noncoding RNA with more than 200 nucleotides (nt) [7]. Previous studies have suggested that lncRNAs play important roles in various cellular and physiological processes, such as chromatin dynamics, gene expression, protein ubiquitination and protein degradation, and glucose metabolism [8,9]. For instance, lncRNAs could induce gene silencing via interaction of histone methylase and histone demethylase, or functionally act as a platform for protein ubiquitination via facilitating E3-ubiquiting ligases assembling [10,11]. In recent years, analysis of transcriptome prolife has revealed that large number of lncRNAs are aberrantly expressed or mutated in various cancers [12]. Accumulating studies also indicated that dysregulated lncRNAs were closely linked to cancer phenotypes including viability, proliferation, growth, motility, immortality, and angiogenesis [7]. To name a few, lncRNA LUNAR1 promoted tumor cell growth via upregulating insulin-like growth factor 1 receptor expression [13]. TGF-β-induced LncRNA ATB facilitated cellular invasion and organ colonization by hepatocellular carcinoma (HCC) cells [14]. Moreover, lncRNAs expression levels are associated with clinicopathological outcomes of cancer patients, such as stratification, metastasis, survival, and recurrence [12,15]. For instance, lncRNA NEAT1, UCA1, and MALAT1 can be used to predict early stage and metastatic lung cancers [16–18]. Also, several systematic reviews have identified a set of upregulated lncRNAs including GHET1, PVT1, and ZEB-AS1 indicated unfavorable survival, worse clinical stage, and metastasis in various cancer patients [19–21]. Therefore, aforementioned evidence concerning lncRNAs correlation with cancer phenotypes and clinical outcomes suggested the potential value of lncRNAs serving as prognostic biomarkers and therapeutic targets in human cancers.

Small nucleolar RNA host gene 12 (SNHG12) is a newly identified lncRNA with aberrant expression in various human cancers [22–24]. Recent published studies have shown that upregulated SNHG12 could drive the tumorigenesis and cancer phenotypes such as proliferation, metastasis, invasion, and anti-apoptosis [25–28]. Furthermore, SNHG12 may serve as a promotor in multiple cancer-related pathways, such as Slug/zinc finger E-box-binding homeobox 2 (ZEB2) pathway, Notch2/Notch pathway, phosphatidylinositol 3-kinase (PI3K)/AKT pathway, and Wnt/β‐catenin signaling pathway [29–32]. Moreover, upregulated SNHG12 expression holds the strong significance on clinicopathological outcomes of cancer patients. Cancer patients with higher SNHG12 expression had unfavorable overall survival (OS), recurrence-free survival (RFS), and disease-free survival (DFS) [28,33–35]. Also, elevated SNHG12 expression was significantly correlated to larger tumor size, positive lymph nodes metastasis (LNM) and distant metastasis (DM), worse clinical stage, and drug resistance [22,31,32,36]. Collectively, SNHG12 has shown its tumorigenesis functions and clinical significance, and may serve as a prognosis biomarker for various human cancers.

However, majority of studies evaluating the prognostic potential of SNHG12 in cancer survival outcomes have been limited by their small sample size and discrete outcomes. In this article, we have reviewed SNHG12 emerging functions and clinicopathological association in multiple kinds of cancers and discussed the potential implication in cancer prognosis. Our work will be the first study using system review methodology to quantitatively evaluate SNHG12 significance on survival and clinicopathological outcomes in human pan-cancers, which would further address the feasibility of SNHG12 as a prognostic candidate in cancers.

## Methods

### Search strategy

We rigorously projected, reviewed, and reported this meta-analysis in line with the PRISMA checklist, and the details of checklist are shown in Supplementary Table 1[37,38]. A systematic literature searching was conducted in several electronic databases, including PubMed, Web of Science, Embase, Scopus, the Cochrane Library, and China National Knowledge Infrastructure (CNKI) and Wanfang databases for eligible studies published by May 25, 2020. The search strategy was as follows: ‘small nucleolar RNA host gene 12 OR SNHG12’ AND ‘cancer OR tumor OR carcinoma OR sarcoma OR malignancy’. Two authors independently completed the literature search, selection, and had discussion to solve any disagreement. Moreover, we checked the citations of retrieved articles for potentially relevant studies.

**Table 1.** Summary of the main characteristics of the studies included in the meta-analysis DFS: disease-free survival; DM: distant metastasis; FISH: fluorescence in situ hybridization; FIGO: the International Federation of Gynecology and Obstetrics; LNM: lymph node metastasis; N/A: not available; NOS: Newcastle-Ottawa Scale; OS: overall survival; RFS: recurrence-free survival; SNHG12: small nucleolar RNA host gene 12; TNM: tumor node metastasis; WHO grade: World Health Organization grade

Study	Year	Tumor type	Sample Size	SNHG12 expression		Follow-up months	Detection assay	Clinical stage	Metastasis analysis	Outcome measure	Survival analysis	Cutoff value	NOS
				High	Low								
Cheng, G et al	2020	Prostate cancer	199	99	100	160	qRT-PCR	N/A	LNM	RFS	Univariate	Median	8
Zhao, G et al	2019	Gastric cancer	56	25	31	60	qRT-PCR	TNM I–IV	LNM/DM	OS	Univariate	Mean	9
Zhang, R et al	2019	Gastric cancer	75	48	27	80	FISH	TNM I–IV	LNM	OS	Multivariate	Mean	9
Wang, X et al	2019	Prostate cancer	56	22	34	60	qRT-PCR	N/A	N/A	OS	Univariate	Mean	9
Song, J et al	2019	Prostate cancer	89	44	45	N/A	qRT-PCR	N/A	LNM	N/A	Univariate	Mean	7
Liu, Y et al	2019	Colorectal cancer	53	26	27	60	qRT-PCR	TNM I–IV	LNM	OS	Univariate	Mean	9
Chen, Q et al	2019	Renal cell carcinoma	20	10	10	108	qRT-PCR	N/A	N/A	OS	Univariate	Mean	7
Zhou, S et al	2018	Osteosarcoma	31	16	15	60	qRT-PCR	Enneking stage	DM	OS	Univariate	Median	9
Zhou, B et al	2018	Osteosarcoma	64	32	32	80	qRT-PCR	N/A	N/A	OS	Univariate	Mean	7
Yang, B et al	2018	Gastric cancer	54	27	27	45	qRT-PCR	N/A	DM	OS	Univariate	Mean	9
Liu, Z et al	2018	Nasopharyngeal carcinoma	129	62	67	60	qRT-PCR	TNM I–IV	N/A	OS	Multivariate	Median	9
Liu, X et al	2018	Glioblastoma	39	31	8	50	qRT-PCR	WHO grade	N/A	OS	Univariate	Mean	9
Lei, W et al	2018	Glioblastoma	79	39	40	60	qRT-PCR	WHO grade	N/A	OS	Univariate	Median	9
Dong, J et al	2018	Cervical cancer	76	38	38	80	qRT-PCR	FIGO stage	LNM	OS	Univariate	Mean	9
Wang, O et al	2017	Breast cancer	102	51	51	N/A	qRT-PCR	TNM I–IV	LNM/DM	N/A	Univariate	Median	7
Wang, J et al	2017	Colorectal cancer	60	30	30	60	qRT-PCR	TNM I–IV	DM	OS	Univariate	Median	9
Lan, T et al	2017	Hepatocellular carcinoma	48	24	24	48	qRT-PCR	TNM I–IV	N/A	OS/RFS	Univariate	Median	9
Zhang, H et al	2017	Gastric cancer	60-	30	30	60	qRT-PCR	TNM I–IV	LNM/DM	OS/DFS	Univariate	Median	9

### Inclusion and exclusion criteria

All eligible studies were critically reviewed and evaluated by two independent investigators (CHZ and XLR). The study would be included in the meta-analysis if it met the following standards: (a) the level of SNHG12 was examined in cancer tissues and adjacent normal tissues; (b) patients were divided into high and low expression groups according to the cutoff value of SNHG12 expression; (c) correlation between SNHG12 expression and survival or clinicopathological features were implicated; and (d) available hazard ratios (HRs) with 95% confidence interval (CI) for OS or RFS could be extracted directly or indirectly.

While the studies meeting following criteria should be excluded: (a) case reports, reviews, letters, meta-analysis and conference reports; (b) irrelevant to human cancer and SNHG12; (c) focused on the function and molecular mechanisms of SNHG12 rather than its association with cancer survival; and (d) animal studies and duplicate publications.

### Data extraction and quality control

Two independent investigators (CHZ and XLR) extracted the following data from each included study: first author name, publication year, tumor type, sample size, number of high SNHG12 expression and low expression groups, follow-up months, detection assay, clinical stage, metastasis, cutoff value, survival outcomes including OS, RFS, and DFS. The missing data regarding survival outcomes was obtained by contacting the corresponding author of eligible articles. If only Kaplan-Meier (K-M) curves were available in the study, the Engauge Digitizer (Version 10.8) was used to synthesize the pooled HRs and corresponding 95%CI via indirect extraction from the curves [39,40]. Since all studies included in this meta-analysis were cohort studies, the study quality was assessed in line with the Newcastle–Ottawa Scale (NOS) by two investigators (WCZ and LQ) [41]. NOS scores ranged from 0 to 9, and studies with score ≥ 6 were considered of high methodological quality. The details of NOS scoring including cohort selection, comparability, and outcome are demonstrated in Supplementary Table 2.

### Online cross-validation in TCGA datasets

We used Gene Expression Profiling Interactive Analysis (GEPIA) to verify the association with OS and DFS and examine SNHG12 expression levels in multiple kinds of cancers. The matched normal data in The Cancer Genome Atlas (TCGA) was used in the validation [42]. The survival analysis was evaluated by Kaplan-Meier method and log-rank test, and the HR and *p* value were shown in the K-M curves.

### Statistical analysis

Extracted data were analyzed by using RevMan 5.3 (The Cochrane Collaboration, Copenhagen, Denmark) and STATA 12.0 (Stata, College Station, TX). Pooled HRs and corresponding 95%CI were utilized to assess the correlation between SNHG12 and prognosis. ORs and 95%CI were applied to evaluate the association between SNHG12 expression and clinicopathological features. Chi square-based Q test and Higgins *I^2^* statistics were employed to determine the heterogeneity across the included studies. *I^2^* value>50% or p-value<0.05 were considered statistically significant and the random-effect model was adopted, otherwise, the fixed-effect model was applied. Sensitivity analysis was conducted by sequentially omitting each single study in order to assess the stability of results. Additionally, Egger`s regression test and Begg`s funnel plot were conducted to evaluate potential publication bias. All p-value were two-sided and a p-value<0.05 was considered significant

## Results

### Characteristics and eligible studies

**Figure 1.**
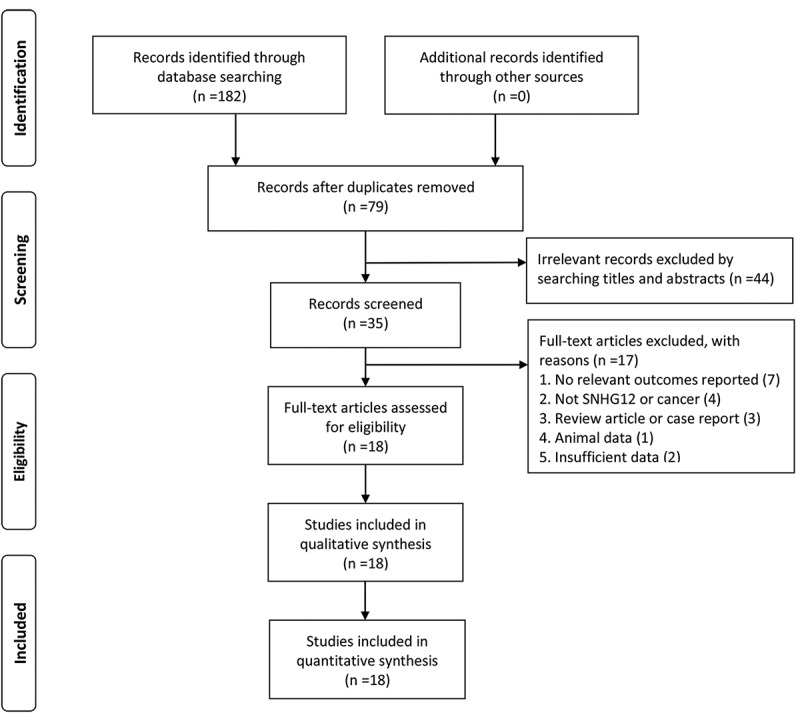
Flow diagram of the literature selection procedure

A total of 182 studies were initially identified as potential articles, and 103 studies were excluded as duplicates. After reviewing titles and abstracts, 44 studies were excluded since they were non-comparative studies or irrelevant topics. Then, 35 potentially eligible articles were selected for full-text assessed, and 17 studies were excluded due to the lack of survival data. Thus, 18 studies compromising 1290 patients were considered eligible in the light of the inclusion and exclusion criteria. The screen procedure was thoroughly implicated via a flow diagram in Figure 1.

The characteristics of the eligible studies are presented in Table 1. These studies were published between 2017 and 2020, and their sample size ranged from 20 to 199. A total of 10 different cancer types were included in our meta-analysis, including prostate cancer, gastric cancer, colorectal cancer, renal cell carcinoma, osteosarcoma, nasopharyngeal carcinoma, glioblastoma, cervical cancer, breast cancer, and hepatocellular carcinoma. Among these 18 studies, quantitative real-time polymerase chain reaction (qRT-PCR) was used as detection assay in 17 studies, and fluorescence in situ hybridization (FISH) analysis was performed in one study. As for survival outcomes, association between SNHG12 expression level and OS were reported in all studies except for three studies only reporting RFS and clinicopathological outcomes, respectively. In all included studies, patients were divided into high or low SNHG12 expression groups according to the cutoff value. Moreover, the follow-up months ranged from 45 to 160 months, and univariate or multivariate analysis were used in survival analysis. As for clinical stage, there were four kinds of clinical stage classification system, including tumor node metastasis (TNM) classification system, the International Federation of Gynecology and Obstetrics (FIGO) stage, Enneking stage, and The World Health Organization (WHO) grade. Additionally, all eligible studies were considered as high methodological quality with their NOS scores ≥7.

### Association between lncRNA SNHG12 and OS/RFS

**Figure 2.**
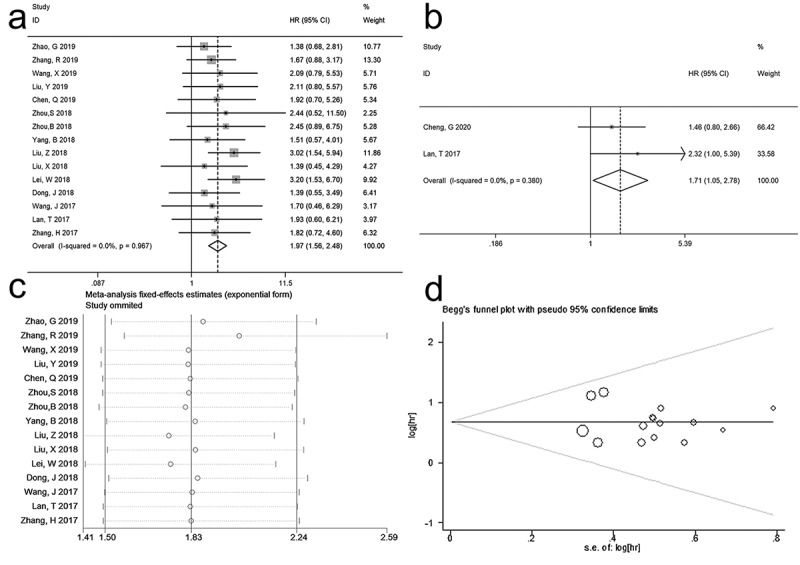
Forest plots of studies assessing the HRs of high SNHG12 expression in human cancers for (a) overall survival and (b) recurrence-free survival. (c) sensitivity analysis of pooled Hazard ratio for overall survival. (d) Begg`s funnel plot for publication bias of SNHG12 on overall survival

A total of 15 studies were included for OS analysis. Since no obvious heterogeneity was observed among these studies (*I^2^ *= 0.0%, p = 0.967), fixed-effects model was employed to synthesize pooled HR and corresponding 95% CI. The aggregated data suggested that high expression level of SNHG12 was significantly correlated to poor OS (HR = 1.97, 95%CI: 1.56–2.48, p < 0.001) (Figure 2(a)), indicating that lower SNHG12 expression in cancer patients may suggest a better survival outcome.

Two studies regarding prostate cancer and hepatocellular carcinoma provided related data for RFS analysis. In the absence of apparent heterogeneity among these studies (*I^2^ *= 0.0%, p = 0.38), fixed-effects model was applied to calculate the HR and its 95%CI. As demonstrated in Figure 2(b), higher SNHG12 expression level indicated unfavorable RFS in prostate cancer and hepatocellular carcinoma (HR = 1.71, 95%CI 1.05–2.78, p < 0.05).

### Sensitivity analysis

Sensitivity analysis was performed in order to assess whether any individual study would affect the result of pooled OS. By removing each included study, we found that the pooled result had a slight fluctuation when ‘Zhang, R 2019’ was removed (Figure 2(c)). Thus, the pooled HR was analyzed again after omitting ‘Zhang, R 2019’, and the result demonstrated that high expression of SNHG12 was still correlated to worse OS in different kinds of cancers (HR = 2.02, 95%CI 1.57–2.59, p < 0.00001, and *I^2^ *= 0.0%, p = 0.957, fixed model), indicating the stability and reliability of this meta-analysis.

### Publication bias

Begg’s funnel plot and Egger’s regression test were employed to evaluate potential publication bias. As shown in Figure 2(d), no apparent asymmetry was observed in the Begg`s funnel plot and the result of Egger’s regression further proved it (p>|t| = 0.160). Therefore, no significant publication bias existed in this meta-analysis.

### Subgroup analysis of association between SNHG12 and OS

**Figure 3.**
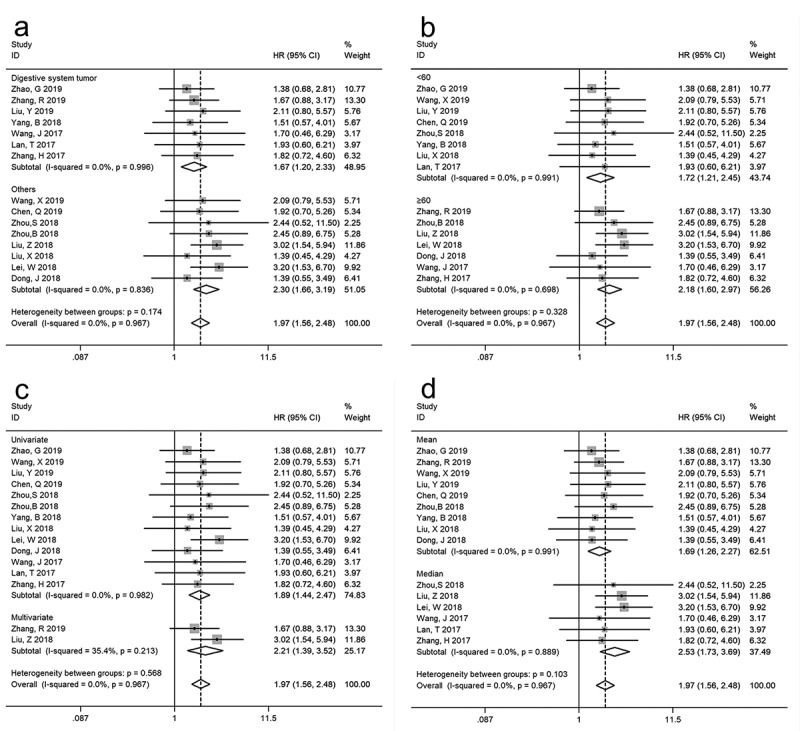
Stratified analyses of SNHG12 expression on overall survival according to subgroups: (a) tumor type, (b) sample size, (c) survival analysis method and (d) cutoff value

Even though the study heterogeneity was low in OS analysis (*I^2^ *= 0.0%, p = 0.967), several stratified analyses were performed based on tumor type (digestive system tumor or others), sample size (more or less than 60), survival analysis method (univariate or multivariate analysis), and cutoff value (mean or median). As shown in Figure 3 and Table 2, all subgroup analyses based on different stratified factors did not alter the association between SNHG12 and OS in multiple kinds of cancers.

**Table 2.** Stratified analyses of the pooled HRs of overall survival by tumor type, sample size, survival analysis method, and cutoff value CI: confidence interval; HR: hazard ratio

			Pooled HR (95% CI)		Heterogeneity	
Subgroup analysis	No. of studies	No. of patients	Fixed model	p-value	I^2^ (%)	p-value
Tumor type						
Digestive system tumor	7	406	1.67 (1.20, 2.33)	0.003	0.0	0.996
Others	8	494	2.30 (1.66, 3.19)	<0.001	0.0	0.836
Sample size						
<60	8	357	1.72 (1.21, 2.45)	0.002	0.0	0.991
≥60	7	543	2.18 (1.60, 2.97)	<0.001	0.0	0.698
Survival analysis method						
Univariate	13	696	1.89 (1.44, 2.47)	<0.001	0.0	0.982
Multivariate	2	204	2.21 (1.39, 3.52)	0.001	35.4	0.213
Cutoff value						
Mean	9	493	1.69 (1.26, 2.27)	<0.001	0.0	0.991
Median	6	407	2.53 (1.73, 3.69)	<0.001	0.0	0.889

### Association between SNHG12 and clinicopathologic characteristics

**Figure 4.**
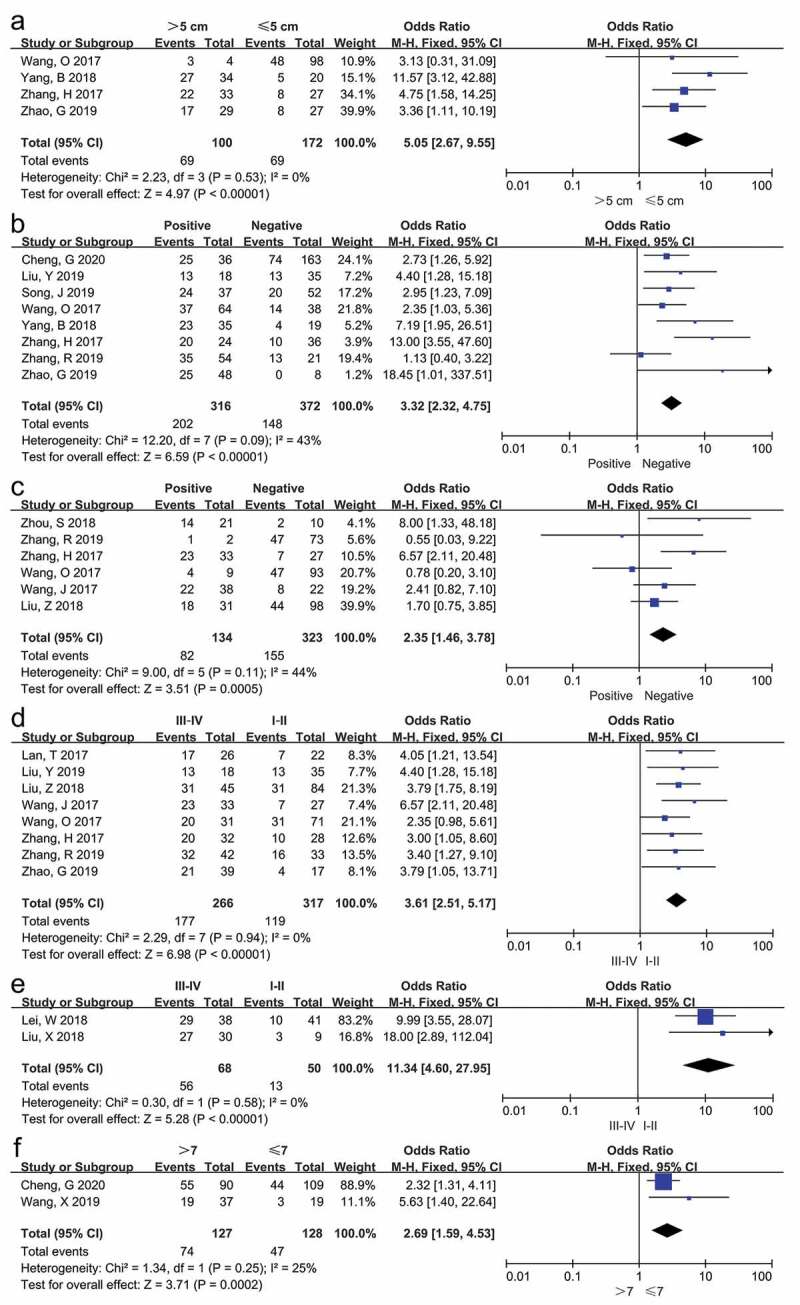
Forest plots evaluating the association between SNHG12 expression and clinicopathological parameters, including (a) tumor size (>5 cm/≤5 cm), (b) lymph node metastasis, (c) distant metastasis, (d) TNM stage, (e) WHO grade, and (f) Gleason score (>7/≤7)

ORs and corresponding 95%CI were applied to investigate the association between SNHG12 and clinicopathologic features including age, gender, tumor size (>5 cm/≤5 cm), Gleason score (>7/≤7), TNM stage, WHO grade, LNM and DM. Fixed-effect model was applied in all analyses and the results of these analyses were implicated in Figure 4, Supplementary Figure 1, and Table 3. Notably, as demonstrated in Figure 4 and Table 3, high expression of SNHG12 had significant association with larger tumor size (p < 0.001), LNM (p < 0.001), DM (p < 0.001), poorer TNM stage (p < 0.001), higher WHO grade (p < 0.001) and Gleason score (p < 0.001). Nevertheless, as shown in Supplementary Figure 1 and Table 3, there was no distinct relationship between SNHG12 expression and age (p = 0.81) or gender (p = 0.96). We could not assess the association between SNHG12 expression and other clinicopathological parameters owing to insufficient data.

**Table 3.** Correlation between lncRNA SNHG12 expression and clinicopathologic parameters for cancers CI: confidence interval; DM: distant metastasis; LNM: lymph node metastasis; OR: odds ratio; SNHG12: small nucleolar RNA host gene 12; WHO grade: World Health Organization grade

						Heterogeneity
Clinicopathologic parameters	No. of Studies	No. of Participants	Pooled OR (95% CI)	p-value	Model	Chi2, p-value, I2 (%)
Age (>60/≤60)	3	191	0.93 (0.51, 1.70)	0.81	Fixed	0.30, 0.86, 0
Gender	11	684	0.99 (0.73, 1.36)	0.96	Fixed	4.86, 0.90, 0
Tumor size (>5 cm/≤5 cm)	4	272	5.05 (2.67, 9.55)	<0.001	Fixed	2.23, 0.53, 0
LNM	8	688	3.32 (2.32, 4.75)	<0.001	Fixed	12.20, 0.09, 43
DM	6	457	2.35 (1.46, 3.78)	<0.001	Fixed	9.00, 0.11, 44
TNM stage	8	583	3.61 (2.51, 5.17)	<0.001	Fixed	2.29, 0.94, 0
WHO grade	2	118	11.34 (4.60, 27.95)	<0.001	Fixed	0.30, 0.58, 0
Gleason score (>7/≤7)	2	255	2.69 (1.59, 4.53)	<0.001	Fixed	1.34, 0.25, 25

### Online cross-validation in TCGA dataset

**Figure 5.**
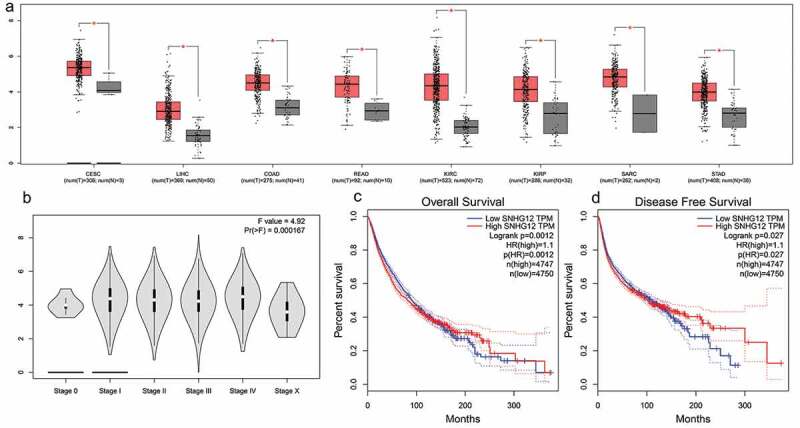
Validation of SNHG12 expression level in multiple cancers in TCGA cohort. (a) The expression level of SNHG12 in breast invasion carcinoma (BRCA), cervical squamous cell carcinoma and endocervical adenocarcinoma (CESC), liver hepatocellular carcinoma (LIHC), colon adenocarcinoma (COAD), rectum adenocarcinoma (READ), kidney renal clear cell carcinoma (KIRC), kidney renal papillary cell carcinoma (KIRP), sarcoma (SARC), and stomach adenocarcinoma (STAD). (b) Violin plot implicating SNHG12 expression levels in different pathological stage of human pan-cancers in TCGA cohort. (c) Overall survival plot of SNHG12 in TCGA cohort (n = 9497). (d) Disease-free survival plot of SNHG12 in TCGA cohort (n = 9497)

We used TCGA dataset to evaluate SNHG12 expression levels in multiple kinds of cancers in order to further validate the pooled results. As depicted in Figure 5, SNHG12 showed aberrant expression levels in cervical squamous cell carcinoma and endocervical adenocarcinoma (CESC), liver hepatocellular carcinoma (LIHC), colon adenocarcinoma (COAD), rectum adenocarcinoma (READ), kidney renal clear cell carcinoma (KIRC), kidney renal papillary cell carcinoma (KIRP), sarcoma (SARC), and stomach adenocarcinoma (STAD) when compared with normal control. Moreover, the violin plot implicated that SNHG12 expression level was significantly correlated with pathological stage in human pan-cancers. Additionally, the survival plots in GEPIA indicated that high expression of SNHG12 predicted worse OS (HR = 1.1, p < 0.05) and DFS (HR = 1.1, p < 0.05), which verified our results in this meta-analysis.

## Discussion

LncRNAs were previously regarded as ‘transcriptional noise’ without any coding effects and did not get much attention among investigators over the past decades [43]. Recently, increasing evidence of next-generation genome wide sequencing and single-cell RNA-sequencing has revealed that lncRNAs have aberrant expressions and mutations in human pan-cancers [44–46]. More and more studies have shown that abnormally expressed lncRNAs are emerging as important regulators in tumorigenesis and show a significant association with cancer prognosis [19,47–49]. SNHG12 is dysregulated in multiple kinds of human cancers, including prostate cancer [22], gastric cancer [31], cervical cancer [34], hepatocellular carcinoma (HCC) [33], renal cell carcinoma [28], nasopharyngeal carcinoma [50], glioma [35], breast cancer [36], non-small cell lung cancer [29], ovarian cancer [24], colorectal cancer [51], and osteosarcoma [30]. Additionally, upregulated SNHG12 expression play important roles in the cellular process of tumorigenesis, including cancer cell proliferation [22,31,50,52], migration [30,33], invasion [28,34], apoptosis [26,27], epithelial-mesenchymal transition(EMT) [29] and chemoresistance [53].

In order to determine the prognostic value of SNHG12 in human cancers, for the first time, we carried out this meta-analysis. The synthesized results implicated that higher expression of SNHG12 indicated worse OS and RFS, and the stratified analyses of OS showed similar results. Moreover, a single study reported that gastric cancer patients with upregulated SNHG12 expression had a worse DFS after surgery [25]. Therefore, SNHG12 overexpression was closely associated with poor survival in cancer patients. The pooled results also showed that patients with higher SNHG12 expression level were more exposed to worse clinicopathological outcomes including larger tumor size, higher Gleason score in prostate cancer, advanced TNM stage, higher WHO grade in glioma, LNM, and DM. In addition, it is worth noting that some clinicopathological parameters only reported in a single study or presented by divergent cutoff values were not included in the pooled results. For instance, prostate cancer patients with high SNHG12 expression were more prone to higher serum prostate-specific antigen (PSA) value, residual tumor, and bone metastasis [22,27]. Osteosarcoma patients with higher SNHG12 expression were more inclined to develop advanced Enneking stage, and vascular invasion occurred more in HCC patients with higher SNHG12 expression [5,30]. Therefore, aforementioned evidence accompanied with our pooled results suggested that high SNHG12 expression level might be an unfavorable biomarker for cancer prognosis. Further, we conducted GEPIA online analyses to validate the prognostic value of SNHG12 in human cancers based on TCGA dataset, and the online validation indicated similar results. Taken together, SNHG12 has the potential serving as a prognostic biomarker in pan-cancer patients.

Even though many studies have indicated the prognostic significance of SNHG12 in human cancers, the further mechanisms remain indistinct. Several investigations have revealed that SNHG12 could function as competing endogenous RNA (ceRNA) by binding to miRNA, thereby regulating target genes in multiple human cancers [22,34]. For instance, SNHG12 upregulation increased the expression of hypoxia-inducible factor 1 α (HIF1α) by targeting miR-199a-5p, which induced cell proliferation, migration, and invasion in renal cell carcinoma [28]. Moreover, doxorubicin resistance in osteosarcoma was promoted by SNHG12 via targeting miR-320a to upregulate myeloid cell leukemia 1 (MCL1) [53]. Similar mechanism was also reported in other human cancers, such as miR-133b or miR-195/cyclin E1 (CCNE1) in prostate cancer [22,27], miR-199a/b-5p/mixed-lineage protein kinase 3 (MLK3) in hepatocellular carcinoma [33], miR-424-5P or miR-125b/signal transducer and activator of transcription 3 (STAT3) in cervical cancer [34,54], miR-16 or miR-320 in gastric cancer [23,25], miR-218/Slug/ZEB2 in non-small cell lung cancer [29], miR-129/SRY-box transcription factor 4 (SOX4) in ovarian cancer [24], miR-16 in colorectal cancer [51], miR-129-5p/WW domain-containing E3 ubiquitin protein ligase 1 (WWP1) in laryngeal squamous cell carcinoma [55], and miR-195/SRY-box transcription factor 5 (SOX5) in glioma [56]. Besides, SNHG12 could be involved into cancer progression by interacting with various kinds of signaling pathways. SNHG12 overexpression promoted cell invasion, migration, and EMT in non-small cell lung cancer via engaging into Slug/ZEB signaling pathway to regulate expression of E-cadherin, matrix metalloproteinase 9 (MMP-9) and vimentin [29]. Similarly, SNHG12 also had cross-talk with other cancer-related pathways including PI3K/Akt pathway and Wnt/β-catenin pathway [31,32]. Considering that SNHG12 has complex function mechanisms in cancers, more studies are still needed to thoroughly explore the association of SNHG12 in different types of cancer.

Recognizing, some limitations to this study should be addressed. First, all the eligible studies were carried out in Chinese population, thus caution must be noticed when applying our results to other population. Second, some HR values were computed via software reconstruction of K-M curves rather than directly obtaining original data, which might lead to bias. Third, the pooled result on RFS should be given caution since only two studies containing hepatocellular carcinoma and prostate cancer were included. Fourth, mean or median value was set as cutoff value in all eligible studies without a consensus standard or detailed description on the calculation process and original data. Thus, the uncertainty about cutoff values across all eligible studies might lead to potential bias.

## Conclusions

Upregulated expression of SNHG12 showed significant association with unfavorable survival and indicated worse clinicopathological outcomes in multiple kinds of human cancer, and therefore might serve as a promising prognosis biomarker and therapeutic target for cancers.

## Supplementary Material

Supplemental Material

## Data Availability

The data used and analyzed in the study is available from the corresponding authors on reasonable request.
