# Supplementary material for: Prognostic and clinical significance of long non-coding RNA SNHG12 expression in various cancers
Source: Bioengineered. 2020 Oct 30;11(1):1112–23. doi: 10.1080/21655979.2020.1831361 (PMC8291808; doi:10.1080/21655979.2020.1831361)
Supplement: Supplemental Material [file KBIE_A_1831361_SM6506.zip › Supp Table 2.docx]

| Study | Total score | Cohort selection | | | | Comparability | Outcome | | |
| --- | --- | --- | --- | --- | --- | --- | --- | --- | --- |
|  |  | Representativeness of the Exposed Cohort | Selection of the Non-Exposed Cohort | Ascertainment of Exposure | Demonstration that outcome of interest was not present at start of study | Comparability of cohorts on the basis of the design or analysis | Assessment of outcome | Was follow-up long enough for outcomes to occur | Adequacy of follow up of cohorts |
| Cheng, G et al | 8 | ★ | ★ | ★ | ★ | ★ | ★ | ★ | ★ |
| Zhao, G et al | 9 | ★ | ★ | ★ | ★ | ★★ | ★ | ★ | ★ |
| Zhang, R et al | 9 | ★ | ★ | ★ | ★ | ★★ | ★ | ★ | ★ |
| Wang, X et al | 9 | ★ | ★ | ★ | ★ | ★★ | ★ | ★ | ★ |
| Song, J et al | 7 | ★ | ★ | ★ | ★ | ★★ | ★ |  |  |
| Liu, Y et al | 9 | ★ | ★ | ★ | ★ | ★★ | ★ | ★ | ★ |
| Chen, Q et al | 7 | ★ | ★ | ★ | ★ | ★ | ★ | ★ |  |
| Zhou, S et al | 9 | ★ | ★ | ★ | ★ | ★★ | ★ | ★ | ★ |
| Zhou, B et al | 7 | ★ | ★ | ★ | ★ | ★ | ★ | ★ |  |
| Yang, B et al | 9 | ★ | ★ | ★ | ★ | ★★ | ★ | ★ | ★ |
| Liu, Z et al | 9 | ★ | ★ | ★ | ★ | ★★ | ★ | ★ | ★ |
| Liu, X et al | 9 | ★ | ★ | ★ | ★ | ★★ | ★ | ★ | ★ |
| Lei, W et al | 9 | ★ | ★ | ★ | ★ | ★★ | ★ | ★ | ★ |
| Dong, J et al | 9 | ★ | ★ | ★ | ★ | ★★ | ★ | ★ | ★ |
| Wang, O et al | 7 | ★ | ★ | ★ | ★ | ★ | ★ | ★ |  |
| Wang, J et al | 9 | ★ | ★ | ★ | ★ | ★★ | ★ | ★ | ★ |
| Lan, T et al | 9 | ★ | ★ | ★ | ★ | ★★ | ★ | ★ | ★ |
| Zhang, H et al | 9 | ★ | ★ | ★ | ★ | ★★ | ★ | ★ | ★ |

**Supplementary Table 2** Study quality and bias in the retrospective cohort studies judged by the Newcastle-Ottawa Scale (NOS) checklist
