# Supplementary material for: Prognostic and clinical significance of long non-coding RNA SNHG12 expression in various cancers
Source: Bioengineered. 2020 Oct 30;11(1):1112–23. doi: 10.1080/21655979.2020.1831361 (PMC8291808; doi:10.1080/21655979.2020.1831361)
Supplement: Supplemental Material [file KBIE_A_1831361_SM6506.zip › Supplementary Figure caption.docx]

**Supplementary Figure 1.** Forest plots evaluating the association between SNHG12 expression and (a) age, (b) gender. The expression of SNHG12 was not correlated with

age(p=0.81) and gender(p=0.96).
